# Early Combination of Material Characteristics and Toxicology Is Useful in the Design of Low Toxicity Carbon Nanofiber

**DOI:** 10.3390/ma5091560

**Published:** 2012-09-03

**Authors:** Ellen K. Jensen, Sten Y. Larsen, Unni C. Nygaard, Calin D. Marioara, Tore Syversen

**Affiliations:** 1 Statoil ASA, Stavanger NO-4035, Norway; 2 Elkem Carbon AS, PO Box 8040 Vaagsbygd, Kristiansand NO-4675, Norway; E-Mail: sten-yngve.larsen@elkem.no; 3 Department of Environmental Immunology, Division of Environmental Medicine, Norwegian Institute of Public Health, PO Box 4404 Nydalen, Oslo NO-0403, Norway; E-Mail: Unni.Cecilie.Nygaard@fhi.no; 4 Department of Synthesis and Properties, SINTEF Materials and Chemistry, Trondheim NO-7465, Norway; E-Mail: calin.d.marioara@sintef.no; 5 Department of Neuroscience, Faculty of Medicine, The Norwegian University of Science and Technology, MTFS, Trondheim NO-7489, Norway; E-Mail: tore.syversen@ntnu.no

**Keywords:** carbon nanofiber, toxicity, characterization

## Abstract

This paper describes an approach for the early combination of material characterization and toxicology testing in order to design carbon nanofiber (CNF) with low toxicity. The aim was to investigate how the adjustment of production parameters and purification procedures can result in a CNF product with low toxicity. Different CNF batches from a pilot plant were characterized with respect to physical properties (chemical composition, specific surface area, morphology, surface chemistry) as well as toxicity by *in vitro* and *in vivo* tests. A description of a test battery for both material characterization and toxicity is given. The results illustrate how the adjustment of production parameters and purification, thermal treatment in particular, influence the material characterization as well as the outcome of the toxic tests. The combination of the tests early during product development is a useful and efficient approach when aiming at designing CNF with low toxicity. Early quality and safety characterization, preferably in an iterative process, is expected to be efficient and promising for this purpose. The toxicity tests applied are preliminary tests of low cost and rapid execution. For further studies, effects such as lung inflammation, fibrosis and respiratory cancer are recommended for the more in-depth studies of the mature CNF product.

## 1. Introduction

Development of new nanomaterials should be accompanied by parallel efforts to investigate and understand their potential health and environmental effects. Lack of epidemiological studies and insufficient toxicological knowledge require a thorough risk management [1,2], covering all aspects of health, safety and environment (HSE). Screening strategies for hazard identification of engineered nanomaterial are given in the literature [3]. Exposure to engineered nanomaterial may occur during the production process, in the subsequent use by companies or consumers, or finally at disposal. In the literature there are few exposure studies from real life. Efforts to make aerosols of carbon nanotubes (CNT), to investigate their relative ease to resuspend, show that this is very difficult [4]. The difficulty to disperse or break up the CNT in order for them to become airborne has been reported [5], pointing out that although various air monitoring systems were run under professional occupational hygiene guidance, no CNT was observed in the air within their laboratory. Newer studies has succeeded in performing real time monitoring of multiwall CNTs [6] and fullerenes [7], respectively, stating their presence in the working atmosphere.

The health effects associated with exposure to CNF (defining CNF as including both CNT and CNF) has been reviewed [8] and since there were no epidemiological studies available, the authors reviewed experimental studies employing both *in vivo* and *in vitro* toxicity models. The conclusion of these authors was that exposure to CNFs in nanomanufacturing plants may represent a possible health risk. A review of CNT exposure assessment and toxicity related to human health indicated that the main risks arose from chronic occupational inhalation, especially during activities involving high CNT release [9]. 

It has been pointed out that toxicological evaluation should be included early in the design of nanomaterials when there are opportunities like modifying synthesis and purification procedures [10]. Inspired by this perspective, we started a research project in 2006 with the aim of investigating how adjustment of production parameters and purification procedures can contribute to engineering nanomaterials of low toxicity. In our case this was CNF, as the companies involved were developing a new reactor in a research pilot plant where one important aim was an efficient production process producing a low toxicity product. At this level, any future commercial application of the CNF product was yet to be developed. CNF has demonstrated its technical usefulness in many of the same application areas as CNTs, e.g., Li-ion batteries [11] and polymer nanocomposites [12].

When the project started in 2006, there were a limited range of *in vitro* toxicity tests which could be applied to non-dissolvable particles. Transferring existing test procedures previously applied to other particles (traffic, indoor dust) proved to be very difficult. The main problems were to generate stable suspensions as particles tended to either float or deposit during the *in vitro* test and secondly the black suspensions interfered with photometric assays. A method was adopted and further developed based on colony formation [13], and this was successfully used to differentiate batches of CNFs produced. 

The *in vivo* tests applied were based on mouse models previously used to identify the allergy-promoting potential of ultrafine particles like carbon black (CB) and diesel exhaust particles [14,15]. The footpad injection model [16] has been found to be a useful hazard identification model for respiratory adjuvants, whereas the intranasal model was applied to investigate the adjuvant effect of particles after exposure via a more relevant route for respiratory allergies. Allergen-specific IgE in serum is a hallmark for allergic diseases and has been used as the main outcome in the *in vivo* studies. Increased levels of allergen-specific IgE may result in an increased risk of developing and/or aggravating allergic symptoms.

The purpose of the material characterization was to allow identification of the particle properties most important for determining the toxicity potential. However, the aim of producing low toxicity CNF shall not compromise the CNF quality beyond an acceptable level, thus particle characterization was also important in order to monitor the product quality. This paper describes test batteries for both material and toxicity testing, and discusses some necessary interventions, *i.e*., homogenization.

## 2. Results and Discussion

### 2.1. Material Characterization

The characteristics of the prepared CNF powders and the reference material CB are shown in Table 1. 

**Table 1:** Characteristics of carbon nanofibers (CNFs) and carbon black (CB).

Name	Thermal Treatment	Surface * (m^2^/g)	D90 ** (µm)	D50 ** (µm)	D10 ** (µm)	S (%)	N (%)	Fe (%)	Si (%)	Ni (%)	P (ppm)	B (ppm)
CNF A	no	103	2.2	1.6	0.7	<0.010	0.017	0.008	0.020	1.29	5.0	0.7
CNF B	yes	61	2.2	1.9	1.6	<0.010	0.016	0.023	0.012	0.070	3.8	2.0
CNF C	no	124	2.6	1.7	0.8	0.095	0.037	0.148	0.056	4.97	8.1	7.6
CNF D	yes	56	2.5	1.5	0.6	<0.010	0.012	0.002	0.004	0.036	0.3	0.3
CB	no	321	6.9	3.2	1.3	0.45	0.121	0.003	<0.03	<0.0003	1.1	<1.0

* BET method (see chapter 3.3.3); ** Coulter counter (see chapter 3.3.2).

As seen in Table 1, both CNF A and CNF C have a high specific surface area compared to CNF B and CNF D, whereas CB displays the highest surface area. It is well known that high temperature treatment of CNF produced by the chemical vapor deposition (CVD) method leads to annealing of micro structural defects as well as reduction of catalytic impurities [11,17]. Formation of energetically stable loops between adjacent active end planes on the surface of annealed CNFs has been described [11]. Such loops have also been observed in the present work, and may contribute to the lower specific surface area observed for the annealed CNFs.

The CNF powders show a similar and narrow particle size distribution. The D90 and D50 value, describing the diameter of the powder below which 90% and 50%, of the distribution lies, give similar results for the various CNFs. CNF B has a higher D10 value compared with the others. The vast majority of the counted CNF diameters (agglomerates) are within the size range of 0.6–2.6 µm. This indicates that the preparation of the powdered CNF samples from the produced batches has been successful in providing powders of similar sizes prior to further investigations. The method does not differentiate between large particles and agglomerates, thus, exact sizes cannot be found from these results. 

The trace elements listed have their origin from the production process of the materials. Thermal treatment reduces the remains from the nickel catalyst, as can be seen for CNF B and CNF D. CNF C is highly contaminated and gives the highest values for all the trace elements listed. The CB differs from the CNF powders in having a higher surface area, a broader agglomerate size distribution (1.3–6.9 µm) and a higher sulfur and nitrogen content.

**Figure 1:**
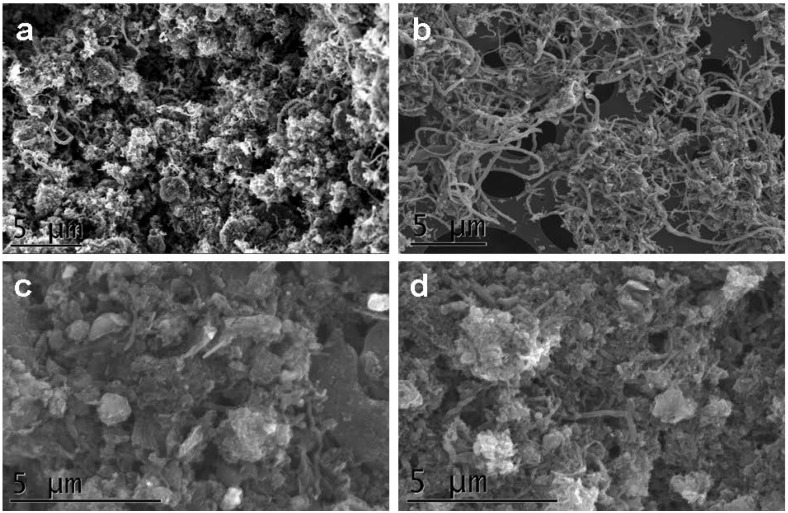
Typical scanning electron microscope (SEM) SE images of the CNF powders, showing (**a**) CNF A; (**b**) CNF B; (**c**) CNF C and (**d**) CNF D.

The scanning electron microscope (SEM) images in Figure 1 show the morphology of the CNF powders. When evaluating the CNFs for fiber content, the CNF B, shown in Figure 1b, is distinctly different from the other three in having the highest fraction of fibers. CNF C (Figure 1c) contains mostly disordered graphitic material and a low fiber fraction. CNF A (Figure 1a) and CNF D (Figure 1d) are somewhere in between the other two batches. The high material quality is a commercially absolute and cannot be compromised, and this is best obtained at the process parameters applied to produce sample CNF B, and poorly obtained in e.g., sample CNF C.

When evaluating whether a fiber is of relevance to exposure or not, it is common to use the WHO (World Health Organization) fiber definition [18]: a fiber is >5 µm long, <3 µm wide, and with a length:width ratio >3:1. Such a fiber can have a relevance to health since its shape enables it to be inhaled and transported within the respiratory system. The definition does not differentiate between fiber types. The CNF samples were checked against the WHO fiber definition by systematically investigate the SEM images with respect to fibers longer than 5 µm. The evaluation was that most fibers had lengths below 5 µm, but estimating number of fibers was not possible since the fibers were highly intertwined.

**Figure 2:**
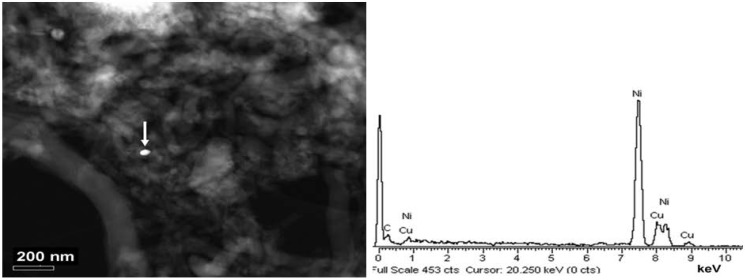
An example of an annual dark field scanning transmission electron microscopy (ADF-STEM) image of sample CNF D, dry powder. The energy dispersive X-ray spectroscopy (EDS) spectrum of the indicated metallic particle is given to the right.

Figure 2 is a typical annual dark field scanning transmission electron microscopy (ADF-STEM) image of CNF D and show that a very low amount of catalyst was present. The metallic particles that were observed were present in clusters and contained Ni and sometimes traces of Fe. The size of the metallic particles varied from below 10 nm to over 50 nm. The low amount of Cu shown in the spectrum is an artifact due to the composition of the grid.

**Figure 3:**
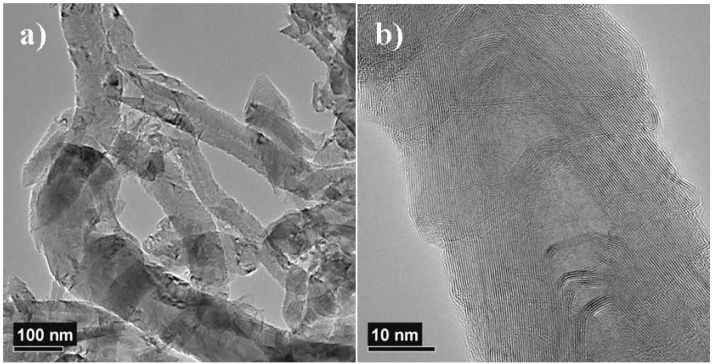

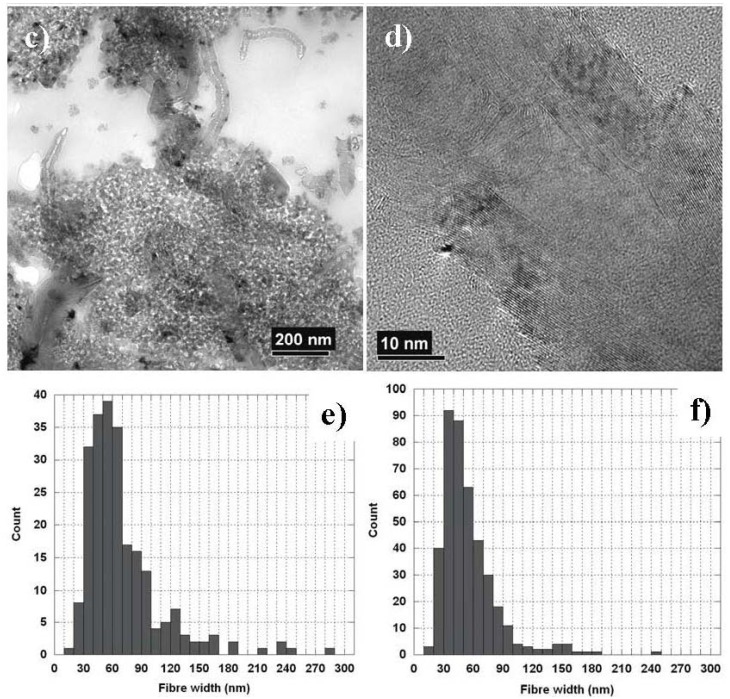
(**a**) A typical transmission electron microscope (TEM) image from sample CNF D dry powder; and (**b**) shows magnification of details; (**c**) A typical TEM image of CNF D distributed in mouse serum; and (**d**) shows magnification of details; (**e**) Fiber width distribution for sample CNF D dry powder; and (**f**) similar for CNF D distributed in mouse serum.

Figure 3 shows typical transmission electron microscope (TEM) images of CNF D. Figure 3a shows intertwined fibers, and the internal fiber structures with carbon atoms ordered as stacks of hats. It can be observed that some fibers are short and appear to be broken, which introduces shorter fiber lengths with “V-shaped” tails. This cutting of fibers in shorter lengths is probably caused by the preparation of CNF powder through mortaring and jetmilling. Figure 3b show a high-resolution image of a typical CNF, which has a periodically closed internal channel. Further, this fiber has a closed loop structure at the surface, and thereby less edge planes available for reactions. This is the result of heat treatment and the removal of contaminants. The possible correlation between the closed loop structure and the lower specific surface area observed for the annealed CNFs, has already been mentioned (Table 1).

Figure 3c shows fiber embedded in crystallized mouse serum, as well as fiber with no serum attached to the surface. The high resolution image given in Figure 3d show that the sonicated fiber has the internal structure intact, but the presence of closed carbon atom layers at the surface is not that clear compared to Figure 3b.

An attempt was made to evaluate the fulfillment of the WHO fiber definition using the TEM images. This failed since these high magnification images did not include the whole fiber length. However, TEM images were well suited for measuring the fiber width, with the width distribution of the CNF D powder sample as an example, presented in Figure 3e. The widths of 231 fibers were measured from 23 TEM images similar to the one shown in Figure 3a. The average fiber width was found to be 71 nm. The smallest width was 19 nm, and the largest was 287 nm.

Figure 3f shows the width distribution of the CNF D sample sonicated in the presence of mouse serum. The width of 411 fibers was measured from 47 TEM images similar to the one shown in Figure 3c. The average fiber width was found to be 55 nm. The smallest width was 15 nm, and the largest was 244 nm. The width distribution of the sonicated mouse serum sample (Figure 3f) shows a reduction in number of thick fibers (width >100 nm) compared to the dry powder sample. This may indicate that the thickest fiber is lost through the sonication procedure.

**Figure 4:**
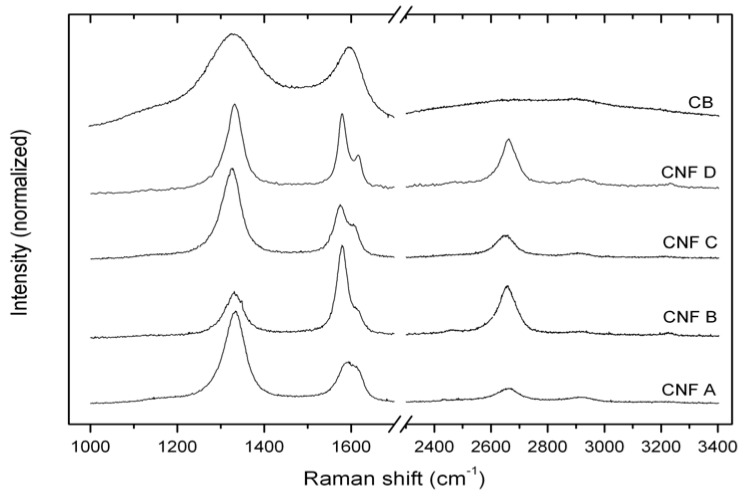
Raman spectra of the CNF powders and CB.

The Raman spectra of the CNFs and CB are shown in Figure 4, where the main features are the usual first-order bands, G and D, at around 1580 cm^−1^ and 1330 cm^−1^, respectively, along with a shoulder at ~1615 cm^−1^ (D’ band) and the 2D overtone at around 2660 cm^−1^ for the CNFs. For the heat treated CNF B and CNF D, a sharper G band along with a more intense 2D band is seen. Following the deconvolution procedure [19], some spectral parameters such as full widths at half maximum (Δν) for the D, G and 2D and the intensity ratios *R* = *ID*/*IG* and *R*’ *R* = *I2D*/*IG* are shown in Table 2. The *R* value [20] showed an inverse relationship to the characteristic coherence length *La*, and has been related to an in-plane coherence length, *L1*, for CNFs and CNTs [19]. The active surface area (ASA) of the samples was calculated from Equation 1. The Raman spectra along with the TEM investigations showed that the CNFs had varying degrees of structural disorder. TEM images of the thermal treated CNFs such as CNF D shown in Figure 3b revealed highly ordered graphene planes, but in contrast to the CNFs, which had not been thermally treated, the graphene planes did not terminate at the edges, but showed a curved “loop” structure. Hence, lower concentration of dangling bonds at the fiber edges are expected for the heat-treated samples, which are also indicated through the lower ASA values.

**Table 2:** Raman data of the CNF powders and CB (equations given in 3.3.3 [21]).

Name	First order lines					Second order lines	
	Δν_D_ (cm^−1^)	Δν_G_ (cm^−1^)	*R = I_D_/I_G_*	*L_1_* (nm)	ASA (m^2^/g)	Δν_D_ (cm^−1^)	*R’ = I_2D_/I_G_*
CNF A	58	42	2.6	3.2	27.1	103	0.4
CNF B	53	29	0.5	17.2	1.6	74	0.5
CNF C	53	34	1.8	4.5	15.2	85	0.4
CNF D	47	24	1.2	7.0	7.4	69	0.6
CB	147	80	1.5	5.4	11.4	314	0.2

**Figure 5:**
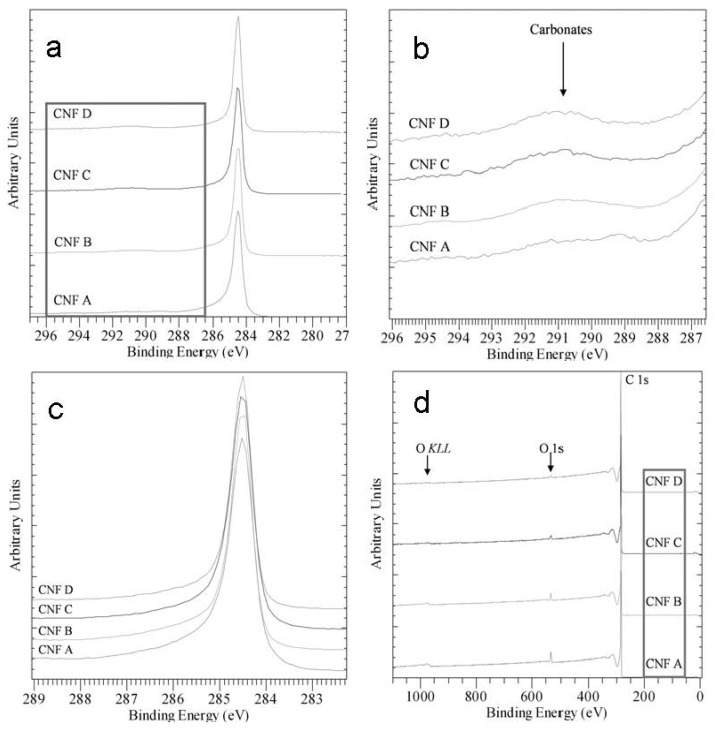

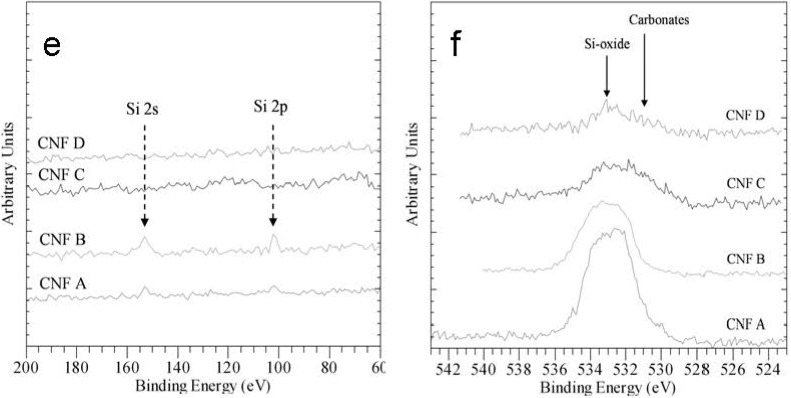
(**a**) High resolution C1s spectra; (**b**) High resolution C1s spectra of the high energy tail (magnification of frame in Figure 5a); (**c**) C1s peaks normalized for approximately the same intensity; (**d**) Survey scans; (**e**) Survey scans of low energy side (magnification of frame in Figure 5d); (**f**) High resolution O1s scans.

Surface chemistry has been characterized with X-ray photoelectron spectrometry (XPS). The main finding is the presence of a low-level Si-oxide contamination on the surface of the carbon-material in samples CNF A and B, see Figure 5. Figure 5a shows that C is present mainly in the C-C chemical state and Figure 5b shows the presence of carbonates. When the C1s peaks are normalized for approximately the same intensity (Figure 5c), no difference is observed between the samples. The survey scans in Figure 5d,e show that samples CNF A and CNF B have higher amounts of O due to the presence of Si-oxide. Figure 5e show that samples CNF A and CNF B contain traces of Si on the surface in oxidized form whereas the surfaces of samples CNF C and CNF D are Si-free. These samples also have lower amounts of O (Figure 5d,f). Figure 5f shows a higher presence of O in samples CNF A and CNF B. The position at ~533 eV corresponds to Si-oxide and/or to adsorbed O, OH species. It is therefore reasonable to assign the peak maxima for samples CNF A and CNF B to Si-oxide, the peak maxima for samples CNF C and CNF D to adsorbed O, OH, and the lower binding energy peak components to carbonates. However, we should point out that the oxygen presence is very low in all samples, resembling typical non-oxidized CNFs [22,23] with samples CNF A and CNF B having higher amounts.

### 2.2. *In Vitro* Toxicity Tests

**Figure 6:**
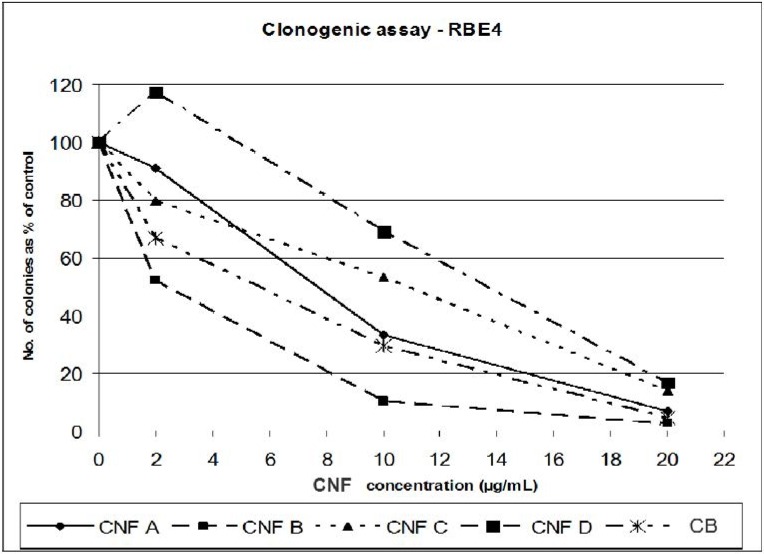
Reduction in RBE4 colony formation after exposure for five days to four different CNF samples. CB included as reference. Bars indicating reproducibility have not been included—please refer to Table 3.

As given in Figure 6, all the tested CNF samples inhibit the formation of RBE4 colonies in a dose-dependent manner. Further details can be found in the study by Gellein *et al*. [13]. The form of the dose-response curve is similar for three of the CNF samples while CNF D shows an initial increase in colony formation at the lowest exposure. The effects in three of the CNFs and CB can be readily observed even at the lowest concentration tested (Figure 6). In Table 3 the same data is given numerically and the standard deviation (SD) is included.

**Table 3:** Number of RBE4 colonies after exposure for five days to four different CNF samples. CB included as reference. Mean and standard deviation are given (n = 3).

Sample	Control		1 (µg/mL)		10 (µg/mL)		20 (µg/mL)	
	Mean	SD	Mean	SD	Mean	SD	Mean	SD
CNF A	64	11.3	59	4.9	21	5.0	4	1.5
CNF B	59	10.6	31	14.0	6	1.7	1	1.0
CNF C	59	10.6	47	10.5	31	3.8	8	2.5
CNF D	62	11.4	73	12.7	43	10.8	10	2.3
CB	59	10.6	39	7.8	17	4.5	3	2.3

The SDs in Table 3 indicate a substantial variation for a given exposure as the relative SD (SD as % of the mean value) range from 8 to 60%—the mean variation being ca. 20%. Thus, this assay needs to be developed further to become better standardized. For comparing samples, which have different dose response patterns, the calculation of EC50 (half maximal effective concentration) can be used as an indicator to differentiate between samples. From the results presented in Figure 6 we have graphically extracted the EC50-concentrations, which are then presented in Table 4. It demonstrates that there are considerable differences in the EC50 for the four CNFs samples tested. Although the few parallel measurements that were possible in this study do not warrant any extensive statistical evaluation, the CNF toxicity appeared to be ranked as CNF B > CNF A > CNF C > CNF D when applying this *in vitro* toxicity test. This pattern did not strongly correlate with changes in any of the process parameter interventions or the material characteristics.

**Table 4:** The effective dose causing a 50% inhibition of cloning (EC50) was determined graphically from Figure 6.

Name	EC50 (µg/mL)
CNF A	7.6
CNF B	2.5
CNF C	10.9
CNF D	13.8
CB	6.6

### 2.3. *In Vivo* Toxicity Test

**Figure 7:**
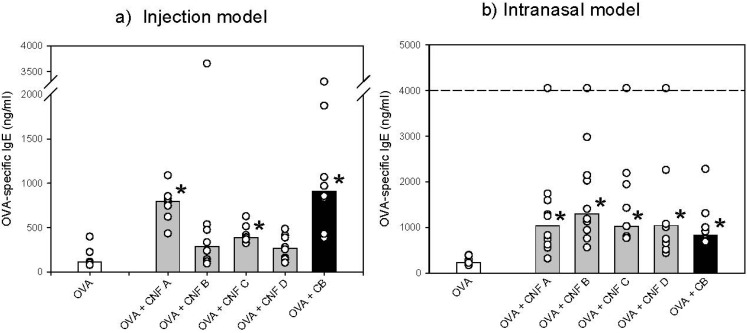
(**a**) Serum levels of OVA-specific IgE on day 26 after subcutaneous injection; and (**b**) intranasal application of OVA alone or together with the four different CNFs, following an allergen booster. Values for individual mice (circles) and median values (columns) for groups of eight and ten mice, respectively, are shown. The dotted line indicates the upper detection limit for the ELISA assay. * Denotes a statistically significant difference compared to the OVA group (p < 0.001), determined by pair-wise comparisons by Tukey’s post hoc test following positive ANOVAs on log-transformed IgE data.

After footpad injection of CNF together with the allergen OVA, only CNF A and CNF C significantly increased the levels of OVA-specific IgE compared to OVA alone (Figure 7a). These results suggested that the four CNF samples differently affected the immune response towards the allergen, depending on the physicochemical characteristics of the CNFs. Interventions in common for CNF B and CNF D, which are the batches less potent with regard to IgE adjuvant effect in the footpad model, are thermally treatment, a process also contributing to changes of particle properties such as reduced metal content and relative surface area.

After exposure via a more relevant route for respiratory allergies, namely intranasal airway exposure, all CNFs significantly increased the OVA-specific IgE levels (Figure 7b), and no differences between the batches were observed. Thus, in the intranasal model, the differences in production process and purification procedure between the CNF batches did not affect this toxicological outcome.

The difference in the IgE adjuvant potency of different particles in the injection and intranasal model has also previously been observed [24], indicating that the airway mucosal lining does modify the immune response towards the particles and allergen. How the different CNFs influence other allergic endpoints or the non-allergic antibody response is currently being investigated. It is important to notice that the IgE adjuvant effect is not necessarily associated with other toxicological endpoints of the airways, such as inflammation, granuloma and fibrosis, which have all been reported after inhalation of CNT in mice [25,26]. Thus, it remains to be determined whether the various CNFs would differ in their putative capacity to induce other biological outcomes *in vivo* than OVA-specific IgE levels. Little is known about how physicochemical characteristics of particles promote IgE production.

### 2.4. Suitability of the Methods

The need for homogenous samples to be used in the subsequent tests (enable representative selection of sample, suitable for *in vitro* and *in vivo* tests) requires the introduction of detailed procedures to process samples. Such procedures may alter the original sample in such ways that the results obtained do not reflect the properties of the original product. The original CNF batches A, B, C and D were slightly changed as a result of the preparation to powdered CNF samples, *i.e.*, removal of the largest agglomerates and longer fibers. This reduction of fiber length as observed in Figure 3a is probably caused by the preparation through mortaring and jetmilling. However, these changes of the original CNF batch seem to occur to the same extent in all samples (CNF A-D) since they show a similar and narrow size distribution (Table 1). The sonication of the sample prior to the toxicity tests add another deviation from the original CNF batch, namely reduction of thick fibers (width > 100 nm). Further, sonication seems to have an influence on the carbon atom layers at the surface as the presence of the loop structure on the outer surface of the heated CNFs becomes less evident. The compromise between the need for a homogeneous sample for the testing and keeping the original qualities of the sample has been continuously challenged. Although the samples applied in the toxicity tests did have a lower content of thick fibers and large agglomerates than the original batch, we find these changes acceptable for our research project.

With regard to exposure and relevance for human health, the highest uncertainty is probably related to dispersion of the CNF in air. In order to be inhaled, the CNFs must be available in the breathing air, and such exposure models are outside the scope of this study. In the *in vivo* tests the mice were exposed through direct injection into the footpad, or through direct placement of a particle suspension on the nostrils. In the *in vitro* test the exposure was through feeding the cells with culture medium containing CNF. Such controlled and artificial exposure situations differ considerably from the real life situation since several of the body defense mechanisms are not available (*in vitro*, injection) or a particle suspension is forced onto the exposure organ (intranasal model). Nevertheless, until real exposure models are available, these toxicity tests may serve the purpose of comparing different types and production batches of similar products. Thus, we may obtain useful data on the relative intrinsic toxicity of such products and an opportunity to guide the development of the production processes. 

The toxicity test applied covers *in vivo* allergy test (immunological response) and an *in vitro* test monitoring the cells’ ability to form colonies, reflecting different mechanisms of toxicity. Both methods have been reported to distinguish between the different toxic potential of particles [13,24]. As preliminary tests, they function well with respect to cost and execution time, and they fit the purpose related to an ongoing development of a production process. They can probably be applied also for other types of solid nanoparticles. However, more specific toxicity tests are needed for studying health effects like lung inflammation, fibrosis and respiratory cancer. Such test are often time consuming but should be applied for the more in-depth toxicity tests of the mature CNF product after the variables of the production process are settled. Even though the CNF batches tested do not fulfill the WHO fiber criteria (most fibers had lengths below 5 µm), based on our results we recommend that the CNF products should be treated with caution and as inhalable particles. 

The direct application of the results from this study is that the production variables for batch B seems promising for further development, since the characterization of CNF B showed the best material quality (highest fiber fraction, low contamination from the catalyst, high degree of crystallinity, limited presence of oxygen on the surface). The *in vitro* toxicity test apparently ranked CNF B as more toxic than the other CNFs tested (Figure 6, Table 4). However, with regard to immunotoxicity, CNF B did not negatively stand out from the other batches in the intranasal *in vivo* tests and appeared to be less toxic in the injection model (Figure 7). Thus, further development of CNF B towards lower toxicity is recommended through fine-tuning of the production variables.

This study illustrates how an approach of early interdisciplinary collaboration is useful during process development, aiming towards manufacturing CNF with low toxicity. Initially, we planned the study as an iterative process in four loops. The first loop consisted of production of a CNF batch, followed by material characterization and toxicological testing, interpretation of the test results, and finally feedback to the production process with respect to adjusting the relevant variables. The next loop could then be performed, and so on, gradually improving quality and reducing toxicity. Unfortunately, we were not able to follow this iterative process within the time frame of the project, since some of the tests took more time to establish than expected. However, when a test battery as presented is available, such a process can be accomplished, and it is expected to be an efficient approach. Future application of early quality and safety characterization, preferably in an iterative design, is promising in order to assure production of CNF with low toxicity.

## 3. Experimental Section

### 3.1. Materials

The four CNF batches (batch A, B, C and D) used in this study were produced in a pilot plant reactor. This process converts natural gas into carbon and hydrogen in a CVD process. The batches represent four different runs of the pilot plant, with differences in variables as gas composition, gas space velocity, nickel catalyst composition, temperature and pressure. Two of the batches, B and D, were taken fresh from the reactor to a thermal treatment. This was performed in a graphite furnace operating under nitrogen atmosphere (2800 °C, 30 min). 

CB (Printex90), kindly provided by Degussa (Köln, Germany) was applied as reference in the study. The relevance of using CB as reference material can be questioned since these particles are spherical while the samples to be tested are fibers. However, CB has been widely used in ultrafine particle studies, is well characterized, is easily available, and is a positive control [27] for the *in vivo* tests applied.

### 3.2. Preparation of Powdered CNF Sample 

The CNF fresh from the reactor (A and C) or from the thermal treatment (B and D) were not suitable as such for the toxicity tests as the size (agglomerates, long fibers) and heterogeneity necessitated a pre-treatment procedure. This preparation started with splitting of the sample using a riffle splitter (Retsch RT 6.5, Retsch Technology GmbH, Haan, Germany), carefully mortaring the sample to pass a sieve (1 mm), and then jetmilling (Alpine 100 AFG fluidized bed opposed jet mill, equipped with classifying plant 50 ATP, Hosokawa Alpine AG, Augsburg, Germany). The carbon powders used in the further study were collected from the filter fraction of the jet mill by using virgin Gore^®^ anti-static polyester felt (475 g/m^2^). Furthermore, the samples were divided into smaller representative samples (approx 0.5 g) using a riffler (Rotary Micro Riffler™, Paul N Gardner Company Inc., Florida, FL, USA). The resulting CNF powders after these preparation steps were applied for the further material characterization and toxicological testing. They were given the names CNF A, CNF B, CNF C and CNF D, respectively, indicating which production batch they originated from.

### 3.3. Material Characterization

The CNF powder samples were then analyzed for several physical characteristics. This included chemical composition (impurities, remaining catalyst), size distribution (relevance for exposure), surface area (area potentially available for contact with human cells), electron microscopy (morphology, dimensions, topological information, internal fiber structure), Raman spectroscopy (structural information), and X-ray photoelectron spectrometry (XPS) (surface chemical composition, functional groups). 

#### 3.3.1. Chemical Composition

The concentration of trace elements was determined by atomic absorption spectrometry (AAS, Varian AA280FS, Agilent Technologies, Santa Clara, CA, USA) and an induced coupled plasma optical emission spectrometer (ICP-OES, Spectro Arcos, Spectro Analytical Instruments GmbH, Kleve, Germany) was used for phosphorus and boron analysis. The sulfur content was determined by a combustion method utilizing IR-absorption of SO_2_ (Eltra CS-2000, Eltra GmbH, Neuss, Germany) and the nitrogen content was determined using the inert-gas fusion principle measuring nitrogen by thermal conductivity (Leco TCH600, Leco Corporation, St Joseph, MI, USA).

#### 3.3.2. Size Distribution

The volume-based particle size distribution of the CNF powders and CB was characterized by using a coulter counter (Beckman Coulter LS 230 analyzer, Beckman Coulter Inc, Brea, CA, USA). Size distribution of the CNF width (diameter) was determined by measurement of individual CNF widths within randomly picked areas of the sample in a transmission electron microscope (Jeol 2010F FEG-TEM, Jeol Ltd, Tokyo, Japan).

#### 3.3.3. Surface Area

The specific surface area was determined by nitrogen adsorption using the Brunauer, Emmet and Teller (BET) method (5-point, Micromeritics Tristar 3000, Micromeritics Instruments Corporation, Norcross, GA, USA) [28,29]. The active surface area (ASA) was calculated using the following empirical Equation [21]:

log [ASA] = 2.3 − 1.7 log *L1* (1)

where *L1* is the in plane coherence length determined by Raman spectroscopy from the intensity ratio, *R* [20]:

*R = I_D_/I_G_* (2)

Using excitation length at 632.8 nm [21] gives the following relation:

*R = I_D_/I_G_ = 8.28/L1* (3)

#### 3.3.4. Scanning and Transmission Electron Microscopy

An identical sample preparation procedure was followed for the scanning and transmission electron microscopy analyses (SEM and TEM, respectively). The dry powders were prepared with ethanol, ultrasound, and dropped on holey carbon film supported by a Cu grid. The test tubes with sonicated serum solutions, as received from the *in vivo* and *in vitro* tests, were shaken by hand and further treated as the others. The SEM analyses were performed with field emission gun (FEG)-SEM (Zeiss Supra 55 VP low vacuum, Carl Zeiss, Oberkochen, Germany) for sample CNF A, and with a different FEG-SEM (Hitachi s-4300se, Hitachi High-Technologies Corporation, Tokyo, Japan) for samples CNF B, CNF C and CNF D. The instruments were operated at 5 and 10 kV. Images were collected at sufficiently low magnifications (3 k–30 k) such that the beginning and end of fibers could be seen. Up to three magnifications were used, with three to four images per magnification. This enabled an estimation of fiber length and morphology. In most cases the secondary electron (SE) and back-scattered electron (BSE) images were recorded from the same area. Images were recorded from at least three to four grid-squares. 

The TEM analyses were performed with a FEG-TEM (Jeol 2010F, operated at 200 kV, image mode point resolution 2 Å). ADF-STEM mode was applied to determine the presence of metallic particles in the analyzed samples. The chemical composition of the metallic particles was investigated with the “Point and ID” option of the INCA software (Oxford Instruments, Oxfordshire, UK) of the Energy Dispersive X-ray Spectroscopy (EDS) system. 

#### 3.3.5. Raman Spectroscopy

Raman spectra of the CNF powders were recorded with a Jobin Yvon LabRam HR spectrometer (Horiba Ltd, Kyoto, Japan) equipped with an Olympus microscope (50 × objective) and He/Ne laser for excitation (632.81 nm). The laser beam power was 0.2–2 mW and each spectral window was recorded up to five times using an integration time of 150–300 sec. First and second order spectra were fitted separately for determination of spectral parameters following a procedure [19] using the OriginPro 8.0 software (OriginLab, Northampton, MA, USA).

#### 3.3.6. Surface Chemistry

The surface chemical composition and functional groups of the CNF powders were analyzed with the XPS technique. The XPS instrument (Kratos Axis Ultra^DLD^, monochromatic Al Kα radiation, h*ν* = 1486.6 eV, 15 kV, 10 mA, Kratos Analytical Ltd, Manchester, UK) used energies of 160 and 10 eV for the survey and the high resolution (C1s and O1s) scans. The powders were positioned in a cup shaped Cu sample holder, and were left overnight in the fast entry chamber to outgas. The initial vacuum in the fast entry lock after positioning the samples was 2 × 10^−6^ torr to 4 × 10^−6^ torr, reaching a value of 2 × 10^−8^ torr next day before performing the experiments. During analysis the vacuum in the analysis chamber was 1 × 10^−9^ torr.

### 3.4. *In Vitro* Toxicity Tests

#### 3.4.1. Sample Suspension

An ultrasonic tip (Ultrasonic processor VCX 750, 750W, Sonics, Sonics and Materials Inc., Newtown, CT, USA) with specified operation parameters (40% amplitude, 30 s each on/off sequential steps, total time 10 min) was employed to disperse the CNF powder in HEPES-buffer (25 mM) fortified with 10% foetal calf serum (FCS, not heat inactivated). The suspensions were prepared by sonicating at an initial concentration (10 mg/mL) of each material. This stock solution was then diluted further by adding the applicable cell culture medium supplemented with 10% FCS to obtain the cell culture exposure concentrations. The suspensions were prepared fresh before every experiment.

During sonication the CNF powder was exposed to energy that may affect its characteristics. Thus, the sonicated serum solution, *i.e*., the stock solution, was characterized with SEM and TEM in order to check for any morphological changes (see Section 3.3.4).

#### 3.4.2. Cell Cultures

Rat brain endothelial cells (RBE4, provided by Dr. Michael Aschner, Vanderbilt University, Nashville, NT, USA) were grown in MEM alpha culture medium (Invitrogen cat 22571-020). The cells require the growth area to be coated with Rat tail Type І collagen (BD biosciences cat. 354236). The media was supplemented with 10% heat inactivated FCS (PAA cat. A15-151), 10 µL/mL penicillin/streptomycin (Gibco cat. 15140-122), 300 µg/mL G418 (Invitrogen cat. 11811-064) and 1 ng/mL basic fibroblast growth factor (Invitrogen cat. 13256-029). All cells were maintained at 37 °C in a humidified incubator at 5% CO_2_.

#### 3.4.3. Cytotoxity Evaluation

Exponentially growing cells were harvested and seeded in 12 well micro plates (Becton Dickinson cat. 353043) at a density of 100 cells/well. Each well contained 1 mL cell culture medium. The cells were allowed to attach for 4 hours. Cells were then treated with CNFs prepared in cell culture medium (1.5 mL) in concentrations of 2 µg/mL (0.52 µg/cm^2^), 10 µg/mL (2.63 µg/cm^2^) and 20 µg/mL (5.26 µg/cm^2^). Three replicate wells were used on each plate for each sample.

After exposure with CNFs for 5 days, the cells were washed twice with PBS, fixed with ice-cold methanol (5 min) and stained with Giemsa solution (Sigma cat. G-5637) for 20 min at 37 °C. After washing with ultrapure water, the number of colonies was determined. Digital photographs were taken of each well and the Nis Elements Advanced Research 2.0 software (Nikon) was used for the evaluation of the digital pictures.

The procedure for the clonogenic assay was adapted from the literature [30,31], and has been successfully used to differentiate between various types of CNTs.

### 3.5. *In Vivo* Toxicity Tests

#### 3.5.1. Sample Suspension

Preparation of the sample suspensions for the *in vivo* tests was performed in line with the preparation performed for the *in vitro* studies with the following adjustments: To the weighed CNF powder, Hank’s balanced salt solution (HBSS; PAA Laboratories GmbH, Linz, Austria) containing the allergen ovalbumin (OVA, chicken egg albumin, grade VII, Sigma, St. Louis, MO, USA) was added to a achieve particle concentrations of 10 mg/mL or 3.8 mg/mL in the injection and intranasal models, respectively. The suspensions were then sonicated (total time 3 min) as described for the *in vitro* sample (Section, 3.4.1). To improve suspension of the particles [32,33] BALB/cA mouse serum (Charles River, Sulzfeld, Germany) was added to a final concentration of 10%, and the suspensions were again sonicated (total time 7 min). The suspensions were diluted in HBSS containing OVA and mouse serum to give the appropriate dose before application in the two models. During sonication the CNF powder was exposed to energy that may affect its characteristics. Thus, the sonicated serum solution, *i.e*., the solution before the final dilution, was characterized with SEM and TEM in order to check for any morphological changes (see Section 3.3.4).

#### 3.5.2. Animals

Female inbred BALB/cAnNCrl mice (Charles River, Sulzfeld, Germany) were housed and kept under conditions previously published [24]. The experiments were performed in conformity with the laws and regulations for experiments with live animals in Norway, and they were approved by the Experimental Animal Board under the Ministry of Agriculture in Norway.

#### 3.5.3. The Footpad Injection Model

To investigate the allergic adjuvant capacity of the CNF, we applied a footpad injection model [16], performed as previously published [24]. In short, the allergen OVA (10 µg) alone or together with the CNF (200 µg), was injected into the right footpad of mice. The mice received an OVA booster injection (10 µg) after three weeks, and five days later the serum levels of OVA-specific IgE were determined by enzyme-linked immuno-sorbent assay (ELISA).

#### 3.5.4. The Intranasal Model

To investigate the allergic adjuvant potential of the CNF in an airway model, mice were exposed intranasally [24]. In short, suspensions of OVA (10 µg) alone or together with CNF (133 µg) were put on the nostrils of anesthetized mice on three consecutive days (resulting in a total dose of 30 µg OVA and 400 µg CNF), followed by intranasal OVA boosters (3 × 10 µg) after three weeks. Five days later, the serum levels of OVA-specific IgE were determined by ELISA.

## 4. Conclusions 

This paper illustrates how combining material characteristics and toxicology testing early in the production process is a useful approach in order to design CNF with low toxicity. CNF material from a pilot plant went through a thorough material characterization (chemical composition, specific surface area, morphology, surface chemistry) and toxicity tests *in vitro* (colony formation) and *in vivo* (immunological responses). When a test battery as presented in this paper is available, early quality and safety characterization, preferably in an iterative design, is expected to be efficient and promising in order to assure production of CNF with low toxicity. The specific results for the four different CNF batches tested showed that thermal treatment of the produced batch was necessary to remove remains of catalyst and metals and to smoothen the surface. The best material quality, *i.e*., high fiber fraction, low contamination from the catalyst, high degree of crystallinity and limited presence of oxygen on the surface, was obtained with CNF B. Thus, further development should be through fine-tuning of the production variables (gas composition, gas space velocity, nickel catalyst composition, temperature, pressure) applied for batch B. The *in vitro* toxicity test apparently ranged CNF B as more toxic than the other CNFs tested. However, with regard to immunotoxicity, CNF B did not negatively stand out from the other batches in the intranasal *in vivo* tests and appeared to be less toxic in the injection model. Thus, further development of CNF B towards lower toxicity is recommended through fine-tuning of the production variables.

The toxicity tests applied may function as preliminary tests having qualities within costs and execution time. Further studies to research the effects such as lung inflammation, fibrosis and respiratory cancer are recommended for the more in-depth studies of the mature CNF product after the variables of the production process are settled.
